# Menstrual Cycle Associated Alteration of Vastus Lateralis Motor Unit Function

**DOI:** 10.1186/s40798-023-00639-8

**Published:** 2023-10-24

**Authors:** Jessica Piasecki, Yuxiao Guo, Eleanor J. Jones, Bethan E. Phillips, Daniel W. Stashuk, Philip J. Atherton, Mathew Piasecki

**Affiliations:** 1 Musculoskeletal Physiology Research Group, Sport, Health and Performance Enhancement Research Centre, Nottingham Trent University, Nottingham, UK; 2 Centre of Metabolism, Ageing and Physiology (COMAP), MRC-Versus Arthritis Centre for Musculoskeletal Ageing Research, National Institute for Health Research (NIHR) Nottingham Biomedical Research Centre, School of Medicine, University of Nottingham, Nottingham, UK; 3 Department of Systems Design Engineering, University of Waterloo, Waterloo, ON Canada

**Keywords:** Menstrual cycle, Motor unit, Estrogen, Progesterone, Electromyography

## Abstract

**Background:**

Estrogen and progesterone are the primary female sex hormones and have net excitatory and inhibitory effects, respectively, on neuronal function. Fluctuating concentrations across the menstrual cycle has led to several lines of research in relation to neuromuscular function and performance; however evidence from animal and cell culture models has yet to be demonstrated in human motor units coupled with quantification of circulating hormones. Intramuscular electromyography was used to record motor unit potentials and corresponding motor unit potential trains from the vastus lateralis of nine eumenorrheic females during the early follicular, ovulation and mid luteal phases of the menstrual cycle, alongside assessments of neuromuscular performance. Multi-level regression models were applied to explore effects of time and of contraction level. Statistical significance was accepted as *p* < 0.05.

**Results:**

Knee extensor maximum voluntary contraction, jump power, force steadiness, and balance did not differ across the menstrual phases (all *p* > 0.4). Firing rate of low threshold motor units (10% maximum voluntary contraction) was lower during the ovulation and mid luteal phases (*β* = − 0.82 Hz, *p* < 0.001), with no difference in motor unit potentials analysed from 25% maximum voluntary contraction contractions. Motor unit potentials were more complex during ovulation and mid luteal phase (*p* < 0.03), with no change in neuromuscular junction transmission instability (*p* > 0.3).

**Conclusions:**

Assessments of neuromuscular performance did not differ across the menstrual cycle. The suppression of low threshold motor unit firing rate during periods of increased progesterone may suggest a potential inhibitory effect and an alteration of recruitment strategy; however this had no discernible effect on performance. These findings highlight contraction level-dependent modulation of vastus lateralis motor unit function over the eumenorrheic cycle, occurring independently of measures of performance.

## Key Points

- Intramuscular electromyography recordings of vastus lateralis motor units (MU) potentials showed a suppression of firing rate of low threshold MUs during the ovulation and mid-luteal phase, with minimal further MU differences identified across three phases of the cycle.
- Measures of knee extensor strength, force steadiness, lower limb power or balance did not differ across the menstrual cycle in healthy young eumenorrheic females.
- Despite differences in the firing rate of low threshold MU, there were no observable effects of the menstrual cycle on neuromuscular performance.

## Background

Estrogen and progesterone are the primary female sex hormones and have the ability to cross the blood–brain barrier and potentially influence the functionality of the central nervous system (CNS) [1]). The fluctuating concentrations of these hormones characterises the menstrual cycle (changing over a period of 22–35 days [2]) which is a natural process for most biological females and serves several physiological maintenance roles beyond reproduction. Although a highly heterogeneous process [3, 4], circulating estrogen typically rises to a peak around day 10–14 followed by a slow decrease over the following 5 days [5]. The latter 14-day luteal phase is characterised by a gradual increase in progesterone, peaking around day 22 and returning to base levels at day 28 [5]. It is unsurprising that the regularly fluctuating concentrations of these hormones with genomic and nongenomic effects has attracted notable research interest in terms of general health [6–9], neuromuscular performance [10–13], and injury risk [14–17].

Estrogen receptors (ERs) are abundant in the CNS and estrogen elicits primarily net excitatory effects via several mechanisms. The ER-α binding of estradiol (E2), the most active type of estrogen, on GABAergic neurons initiates the suppression of GABA (the primary inhibitory neurotransmitter) via destabilisation of GABA receptors minimising inhibitory action in cultured neurons and hippocampal sections [18]. E2 supports the potentiation of the effects of excitatory glutamatergic neurons [19] and promotes glutamate receptor trafficking [20]. E2 has also been shown to affect the signalling of the neurotransmitter serotonin throughout the CNS which may have downstream effects on motoneuron firing rates [21–23]. Opposingly, progesterone has a net inhibitory effect, shown to cause a decrease in discharge rate of Purkinje neurons during locomotion in animal models [24] and increased inhibition of rat pyramidal neurons [25]. Progesterone also reduces the availability of estrogen specific receptors on neuronal cells, enhances the inhibitory responses of limbic neurons [19], suppresses the excitatory response of glutamate [26] and increases GABA release [27–29]. Although direct excitatory and inhibitory effects of these hormones have been well described, it is important to note a net neuronal effect in complex physiological processes (e.g. human performance) is a result of factors beyond directly measurable estrogen and progesterone alone.

In addition to influencing neural excitability, estrogen may also influence neuromuscular performance directly at the muscle fibre, potentially enhancing myosin binding [30]. In smooth muscle, estrogen can acutely inhibit L-type Ca^2+^ channels and limit contractility [31], which are also located at pre and post-synaptic regions of the neuromuscular junction (NMJ) and may exert similar inhibitory effects there via interference of acetylcholine release and receptor binding [32]. Motor unit (MU) firing rate is a key determinant of force generating capacity and may be susceptible to the inhibitory/excitatory effects of sex hormones. Of the only study to investigate this, alterations of vastus medialis MU initial discharge rate were reported across 5 stages of the menstrual cycle, which may have corresponded with fluctuating estrogen and progesterone [10]. Here, it was postulated that progesterone may have the more dominant effect on MU firing, although this was not quantified [10]. Collectively, these studies provide strong indications of the acute excitatory and inhibitory effects of sex hormones.

Although physiologically plausible, the lack of in- or ex-vivo data at central and peripheral sites of the motor system is further complicated by the general lack of female data [33] and the equivocal findings of the menstrual cycle on neuromuscular performance, with some reporting greater strength at the menstrual phase [34], the follicular phase [35], at the mid-point of the cycle [11, 36], and some reports of increases at the luteal phase [37]. Several others report no change in performance across the cycle [38–42], yet 36–51% of athletes identify their menstrual symptoms to adversely affect their performance [43, 44]. Importantly, recent systematic review and meta-analyses have highlighted a lack of consistent high-quality research, alongside a largely trivial difference in strength across a eumenorrheic cycle [45, 46].

There are currently no data quantifying MU adaptation combined with menstrual tracking and hormone quantification across the human menstrual cycle. Therefore, the purpose of the present study was to assess neuromuscular performance and quantify vastus lateralis (VL) MU function using intramuscular electromyography (iEMG) at different contraction intensities over three key stages: early follicular, ovulation, and mid luteal phases of a single menstrual cycle in healthy eumenorrheic young females.

## Methods

### Participant Information

The research study was approved by local ethics committees at the University of Nottingham (302-1903). The study conformed with all standards set by the Declaration of Helsinki, except for registration in a database. A total of 13 recreationally active (defined as meeting World Health Organisation minimum activity guidelines [47], and not regularly competing in sports) females volunteered to take part in this research study. Three participants were unable to attend the second visit within the required ovulation window, and one participant was excluded based on low and non-changing progesterone levels. Full data are presented for nine females with a mean (standard deviation: SD) age of 24.2 (3.2) years and a BMI of 22.8 (2.8). Exclusion criteria included a diagnosis of metabolic disease, lower limb musculoskeletal abnormalities, acute cerebrovascular or cardiovascular disease, active malignancy, uncontrolled hypertension or those on medications that are potentially neuroactive or modulate vascular tone. All participants were classified as eumenorrheic having reported a regular cycle between 21 and 35 days [2] for the previous 12 months and had not taken any oral contraceptive pill within the previous 12 months. Participants arrived at the laboratory after an overnight fast, and body mass and height were measured on the first visit only. The three testing sessions occurred at the same time of day for each stage of the cycle, and participants refrained from strenuous exercise and alcohol for 24 h prior. To consider the influence of the menstrual cycle on neuromuscular performance participants were assessed at three time points across the cycle; (1) Early follicular; within 48 h of the onset of the menstrual period. (2) At ovulation; determined by home-based ovulation kits (One Step, AllTest BioTech Co Ltd, Germany. Ovulation accuracy deemed > 99.9%) used to detect the rise in luteinizing hormone associated with ovulation. Upon a positive test, participants were tested in the laboratory within 48 h. (3) The mid luteal phase; assessed 7 days following ovulation [48].

### Plasma Hormones

Two ethylenediamine tetra-acetic acid (EDTA) tubes (20 ml) of blood were drawn from the antecubital vein at each visit. All tubes were centrifuged at 3200*g at 4 °C for 20 min. Plasma was then aliquoted into 1 ml Eppendorf tubes and stored at − 80 °C for future analysis. Stages of the menstrual cycle were confirmed via determination of the plasma concentration of 17β-estradiol and progesterone. Analysis of plasma samples was conducted using enzyme-linked immunoassay kits (Invitrogen, Thermofisher Scientific, Camarillo, CA) following manufacturer’s instructions, measured at an absorbance of 450 nm. The minimal detection concentration of 17β-estradiol was 5 pg mL, and progesterone was 4 pg mL. Each sample was added to the ELISA panel in duplicate, and a standard curve plotted with 6 standards for 17β-estradiol and 8 standards for progesterone. The concentration of hormones within the plasma samples was then determined from the mean absorbance of the duplicates, interpolating directly from the standard curve. The Coefficient of Variation (CoV) for 17β-estradiol was 3.9–6.1% and for progesterone 3.5–7.0%. Participant hormonal samples all met previously recommended criteria [49] when conducting experimental procedures across the menstrual cycle, with a peak in progesterone concentration at the mid-luteal phase and a rise in 17β-estradiol from early follicular to ovulation. One participant had a progesterone concentration of 0.9–1.0 ng ml at all time points and was not included in final analysis.

### Neuromuscular Performance

Participants were seated in a custom-built chair with hips and knees flexed at 90°. The lower leg of the right limb was securely attached to a force dynamometer with non-compliant straps (purpose-built calibrated strain gauge, RS125 Components LTD, Corby, UK) above the medial malleolus. Participants were also strapped into the chair with a seat belt across the pelvis to minimise movement of the upper trunk throughout testing. All participants underwent a standardised warm up of submaximal isometric contractions. Participants were instructed to only perform these sub-maximal contractions as a familiarisation to the procedure, no maximum contractions were performed as part of the warm up. Once prepared and instructions fully understood, participants were then instructed to perform a maximum voluntary contraction (MVC), pushing as hard as possible, receiving visual feedback and verbal encouragement (e.g. “push, push”) from the researchers. This was repeated a total of 3 times, with 60 s rest between each repetition, taking the highest values, in Newtons, to be the accepted MVC. All participants achieved a plateau in peak force by the third effort; therefore, it is certain the maximum effort was achieved.

After the MVC, participants were given a minimum 2-min recovery before proceeding with the force steadiness task. Prior to the force steadiness assessments, participants conducted a familiarisation, matching a target force on a screen, for around 12–15 s each time, at 10%, 25% and 40% MVC. Once familiarised, participants were instructed to perform the experimental contractions, 4 sustained contractions at 10 and 25% MVC, and 2 at 40% MVC for 12–15 s each, the order of which was randomised, with 30 s rest between each contraction to ensure no muscle fatigue was acquired. All participants were able to achieve the required level of force output.

An RS Foot scan (Gait and Motion Technology Ltd, Bury St Edmunds, UK) pressure sensor plate was used to assess single leg balance. The centre of pressure (COP), or postural sway, of the vertical plane was measured throughout assessment and is expressed as total distanced moved in millimetres (mm). This was assessed for 30 s with participants standing in the centre of the sensor plate, on the right leg only. To assess jump power, a G-walk sensor was placed at the base of the spine, secured with a Velcro waist belt (Gait and Motion Technology Ltd). Participants were asked to perform a counter movement jump, instructions were to jump as high as possible, with hands remaining on their waist with a trained assistant present and in reach of the participants in case of a fall or falter. Each participant repeated the jump sequence three times, with approximately 30 s rest between jumps, and the highest value was recorded [50].

### Intramuscular Electromyography (iEMG)

A 25-mm disposable concentric needle electrode (Ambu Neuroline, Ambu, UK) was used for all iEMG assessments. The needle was inserted into the muscle belly of the VL in the area of the motor point, established as previously described [51]. iEMG signals were recorded using Spike 2 (Version 9.06) sampling at 50 kHz and bandpass filtered at 10 Hz to 10 kHz (1902 amplifier; Cambridge Electronic Design Ltd, Cambridge, UK) and stored for future off-line analysis. A ground electrode was placed over the patella. Prior to sampling, participants were instructed to perform a series of voluntary low-level contractions with the needle in place to ensure adequate signal-to-noise ratio (e.g. visible spikes). Participants then performed sustained voluntary contractions as detailed above. After performing contractions at each contraction level the needle electrode was repositioned with combinations of turning the bevel 180 degrees and withdrawing by ~ 5 mm. This was repeated to perform 4 contractions recording from spatially distinct areas [52]. Participants had 30 s rest between each contraction.

### iEMG Analysis

Decomposition-based quantitative electromyography (DQEMG) software [53] was used to detect motor unit potentials (MUPs) and their corresponding motor unit potential trains (MUPTs). MUPTs composed of MUPs from more than one MU or with fewer than 30 MUPs were excluded from further analysis. Template MUPs of all MUPTs were visually checked, with markers adjusted where necessary to correspond to the onset, end, and positive and negative peaks. All MU data were analysed from the sustained phase of the contraction only, excluding rise and fall phases. iEMG data are reported for 10% and 25% MVC contractions only as reliable MUP identification was not possible for all participants at 40% MVC.

MU Firing rate (FR) was assessed as the rate of MUP occurrences within a MUPT, which is expressed as the number of occurrences per second (Hz). The MU FR variability is reported as the CoV for the interspike interval (ISI). Area of the MUP was measured as the total area within the MUP duration (from onset to end). MUP complexity is reported as the number of turns, measured as a change in waveform direction of at least 25 µV, indicating the level of temporal dispersion across individual muscle fibre contributions to a single MUP. A near fibre MUP (NFM) is defined as the low-pass-second derivative [54] of its corresponding MUP and is primarily contributed to by fibres within 350 µm of the recording surface of the electrode. This ensures that only potentials from fibres that are nearest to the needle electrode contribute to a NFM and that NFMs are composed of reduced contributions from more distant MU fibres and less interference from distant active fibres of other MUs. Any NFMs that did not have clear peaks were excluded from subsequent analysis. NFM jiggle is a measure of the variability of the shape of consecutive NFMs of an MUPT reflective of NMJ transmission instability, and are expressed as a percentage of the total NFM area [54].

### Statistical Analysis

The Shapiro–Wilk test was used to examine data distribution using GraphPad Prism (V 9.4.1). Where normally distributed, univariate ANOVA was performed to identify any differences between hormone levels, MVC, jump power, balance performance and force steadiness (FS) at 10 and 25% MVC, followed by Tukey’s post hoc test. Where not normally distributed (FS at 40% MVC only), Friedman test was applied. Estimated mean differences (EMD) are reported with any statistically significant repeated measures comparisons. For MU characteristics, mixed-effect linear regression analysis was performed using the *lme4* package (V 1.1.23) [55] in R (V. 4.1.2), using RStudio (V. 2022.12.0.353). Models were generated to compare the three timepoints across the menstrual cycle at each contraction level, with timepoint as fixed effect, and then separately to compare differences with increasing contraction level (i.e. from 10 to 25% MVC) across stages, with contraction level as a fixed effect. Participant was included as a random effect in all cases (e.g. (1|Subject). The linear mixed-effect modelling is appropriate for data of this nature as it incorporates the whole sample of extracted MUPTs, rather than just the mean values that were obtained from each participant and provides coefficient estimates that indicate magnitude and direction of effects of interest. Results of these outputs are displayed as *β* coefficient estimates, 95% confidence intervals and p-values, for *N* = 9. Standardised betas and 95% confidence intervals are also shown for each model output. To explore the association between sex hormones and MU characteristics, separate linear models were generated for estrogen and progesterone, using participants means for each MU characteristic, with time as a fixed effect. Statistical significance was assumed when *p* < 0.05.

## Results

**Fig. 1:**
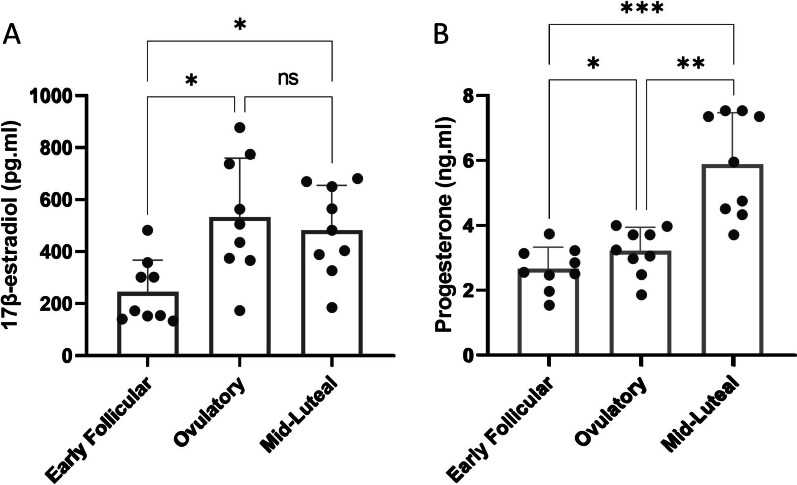
Circulating concentrations of **A** estrogen (17β-estradiol) and **B** progesterone across menstrual cycle phases. *p < 0.05; **p < 0.01; ***p < 0.001. *ns* Non-significant

Estrogen concentrations differed across the 3 time points (*p* = 0.002), with a mean of 244 pg ml in the early follicular phase, rising to 533 pg ml in the ovulatory phase (*p* = 0.016, EMD = 289). At 484 pg ml in the mid-luteal phase, this was significantly higher than the early follicular (*p* = 0.013, EMD = 239) but not the ovulatory phase (*p* = 0.744, EMD = 50.5) (Fig. 1A). Progesterone also differed across the 3 time points (*p* < 0.001); with a mean of 2.66 ng.ml in the early follicular phase, rising to 3.22 ng.ml in the ovulatory phase (*p* = 0.027, EMD = 0.556), and rising again to 5.89 ng.ml in the mid-luteal phase (*p* = 0.008, EMD = 2.667), which was significantly higher than the early follicular phase (*p* < 0.001, EMD = 3.225) (Fig. 1B).

**Fig. 2:**
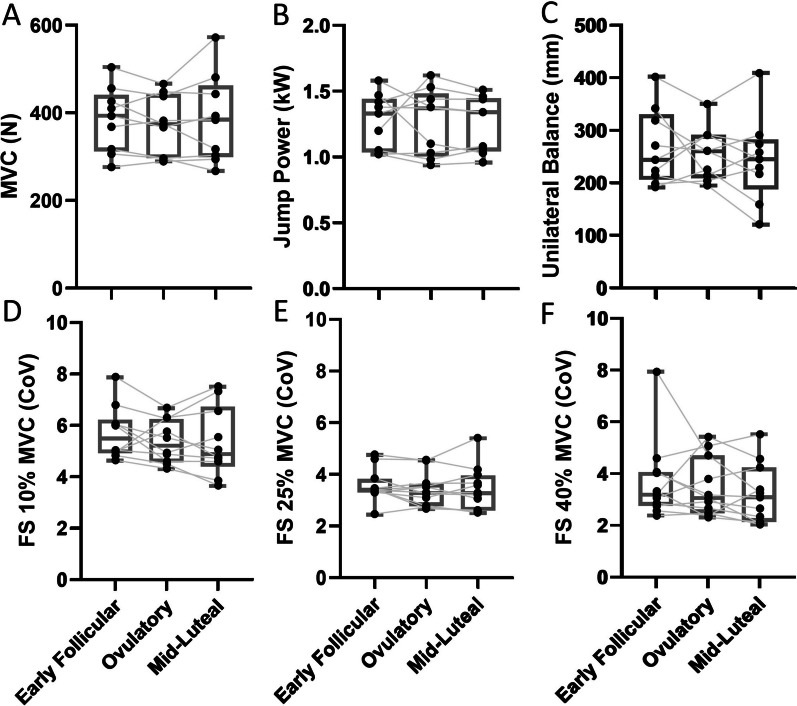
Knee extensor maximal isometric strength (**A**), jump power (**B**), right leg single balance (**C**), knee extensor force steadiness at 10% (**D**), 25% (**E**), 40% MVC (**F**), assessed at early follicular, ovulatory and mid-luteal phases of the menstrual cycle. *MVC* Maximum voluntary contraction, *N* Newtons, *W* Watts, *mm* Millimetres, *FS* Force steadiness, *CoV* Coefficient of variation

There were no significant time-associated differences in MVC (*p* = 0.624), jump power (*p* = 0.775), or distance travelled during right leg unilateral balance (*p* = 0.713) (Fig. 2A–C). Similarly, force steadiness did not differ across the cycle when assessed at 10% MVC (*p* = 0.453), 25% (*p* = 0.405), or 40% MVC (*p* = 0.813) (Fig. 2D–F).

**Fig. 3:**
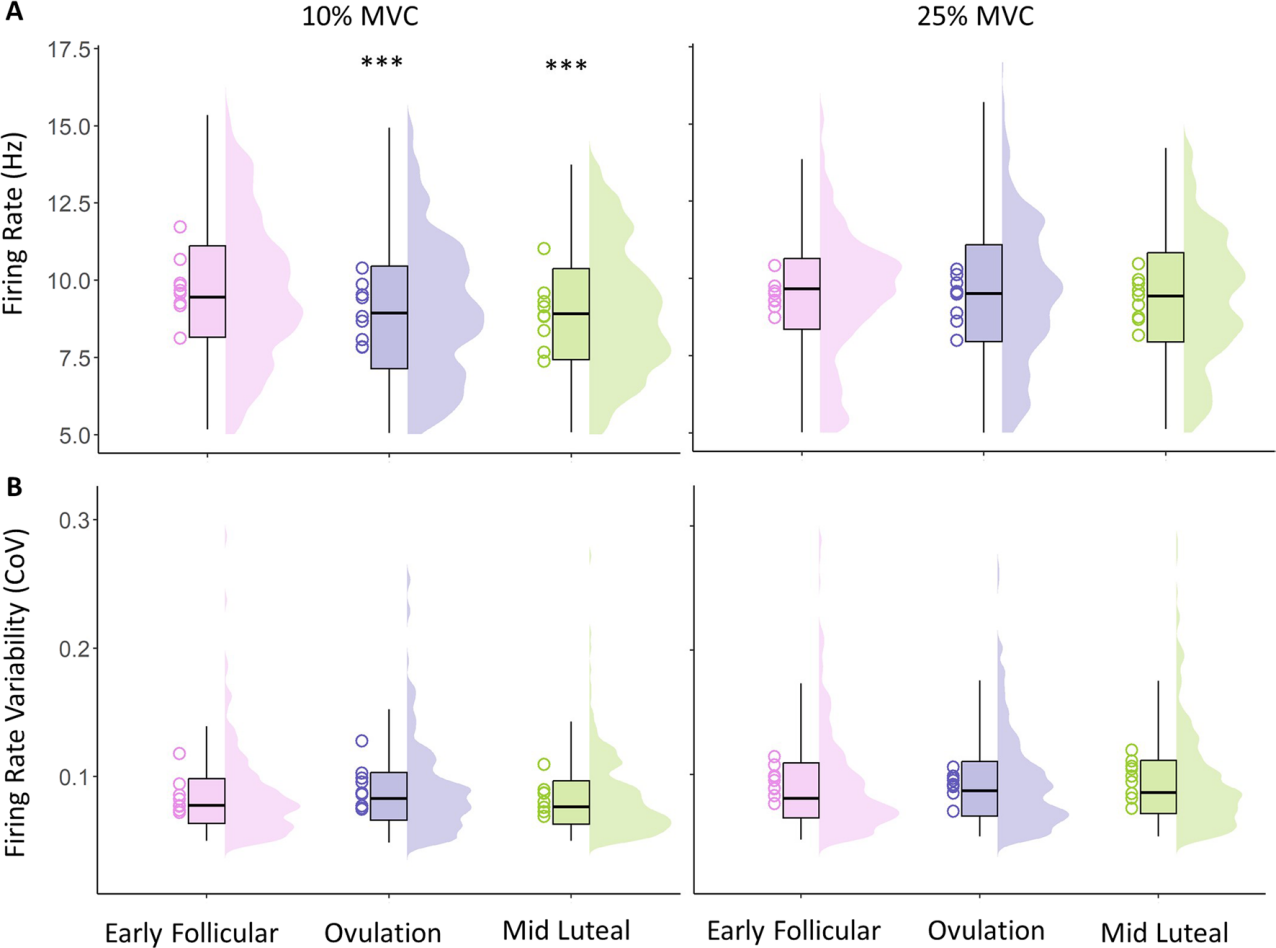
Motor unit firing rate (**A**) and firing rate variability (**B**) recorded at 10% and 25% MVC, at 3 stages of the menstrual cycle. Data show individual participant means as hollow circle, with all analysed MUPTs shown in boxplots and distribution plots. *MVC* Maximum voluntary contraction, *CoV* Coefficient of variation. Comparison to EF shown as ***p < 0.001

At 10% MVC a total of 694 MUPTs were analysed in all participants, with a total of 201 (~ 22 per participant) in the early follicular phase, 239 (~ 26 per participant) in the ovulatory, and 254 (~ 27 per participant) in the mid-luteal phase. At 25% MVC a total of 939 MUPTs were analysed in all participants, with a total of 326 (~ 35 per participant) in the early follicular phase, 307 (~ 36 per participant) in the ovulatory, and 306 (~ 34 per participant) in the mid-luteal phase. Individual means and all MU data reflecting firing rate and firing rate variability at 10 and 25% MVC are shown in Fig. 3A, B, with statistical outputs from multi-level models shown in Table 1. At 10% MVC, multi-level models revealed a lower MU firing rate at ovulation and mid luteal phases when compared to the early follicular phase (both *p* < 0.001), and no difference between ovulation and mid luteal phases (*p* = 0.792). Firing rate variability did not differ across any of the 3 phases. At 25% MVC, MU firing rate nor firing rate variability differed across the menstrual cycle (all *p* > 0.3) (Table 1).

**Table 1:** Multilevel model analysis summary for motor unit parameters across three points of the menstrual cycle, at 10% and 25% MVC

	10% MVC			25% MVC		
	Beta	95 CI	*p* value	Beta	95 CI	*p* value
Firing rate (Hz)						
* EF v Ov*	− **0.804**	− **1.206 to** − **0.402**	** < 0.001**	0.005	− 0.341 to 0.352	0.976
*EF v ML*	− **0.855**	− **1.255 to** − **0.456**	** < 0.001**	− 0.110	− 0.464 to 0.245	0.546
*Ov v ML*	− 0.051	− 0.432 to 0.330	0.792	− 0.114	− 0.474 to 0.244	0.532
Firing rate variability						
*EF v Ov*	0.005	− 0.002 to 0.010	0.146	− 0.001	− 0.008 to 0.005	0.692
*EF v ML*	− 0.003	− 0.009 to 0.004	0.376	0.002	− 0.005 to 0.009	0.540
*Ov v ML*	− 0.007	− 0.013 to − 0.001	0.078	0.003	− 0.003 to 0.010	0.323
MUP area (µV ms)						
*EF v Ov*	− 70.79	− 142 to 0.53	0.059	− 27.64	− 100.8 to 45.6	0.460
*EF v ML*	32.19	− 39.62 to 104.1	0.381	70.44	− 3.05 to 143.9	0.061
*Ov v ML*	**102.9**	**35.19 to 170.78**	**0.003**	**98.09**	**23.1 to 173.1**	**0.031**
MUP turns						
*EF v Ov*	0.095	− 0.243 to 0.433	0.582	− 0.031	− 0.255 to 0.193	0.788
*EF v ML*	**0.604**	**0.264 to 0.944**	**0.001**	**0.385**	**0.159 to 0.610**	**0.001**
*Ov v ML*	**0.509**	**0.188 to 0.830**	**0.003**	**0.415**	**0.186 to 0.645**	**0.001**
NMJ transmission instability (%)						
*EF v Ov*	0.283	− 1.94 to 2.51	0.804	0.221	− 2.01 to 2.46	0.846
*EF v ML*	− 1.029	− 3.15 to 1.09	0.345	− 0.475	− 2.85 to 1.35	0.490
*Ov v ML*	− 1.311	− 3.45 to 0.83	0.688	− 0.966	− 3.15 to 1.22	0.999

Model outputs show unstandardised beta, 95% confidence interval and associated *p* values

*MVC* Maximum voluntary contraction, *EF* Early follicular, *Ov* Ovulation, *ML* Mid luteal, *MUP* Motor unit potential, *NMJ* Neuromuscular junction

Bold indicates significantly different

**Fig. 4:**
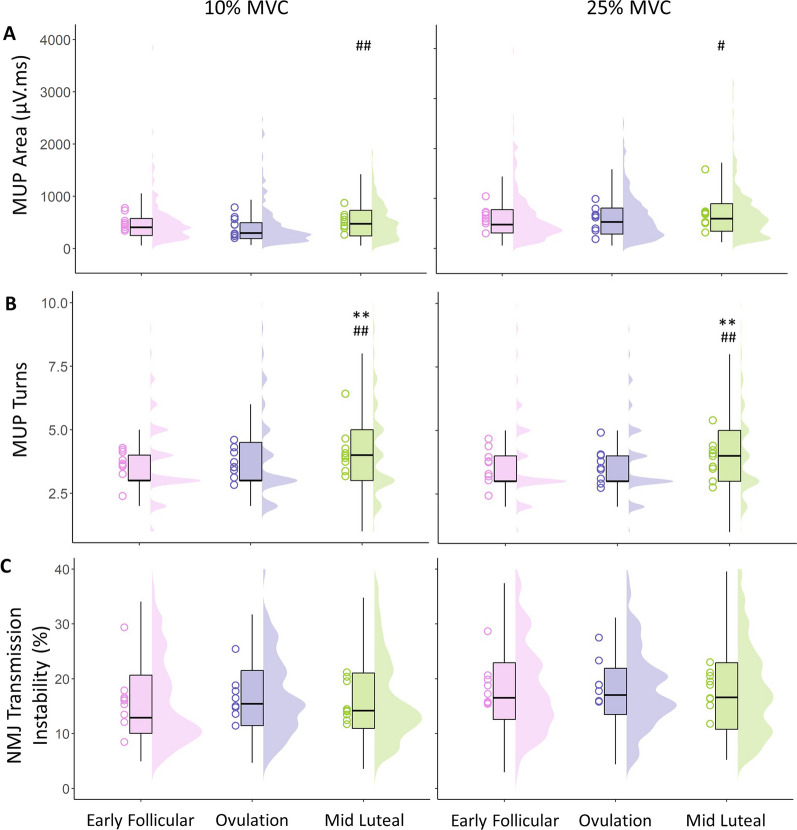
Motor unit potential area (**A**), complexity (number of turns) (**B**), and NMJ transmission instability (**C**) recorded at 10% and 25% MVC, at 3 stages of the menstrual cycle. Data show individual participant means as hollow circle, with all analysed MUPTs shown in boxplots and distribution plots. *MVC* Maximum voluntary contraction, *CoV* Coefficient of variation. Comparison to Ov shown as ^#^*p* < 0.05, ^##^*p* < 0.01. Comparison to EF shown as **p < 0.01

**Fig. 5:**
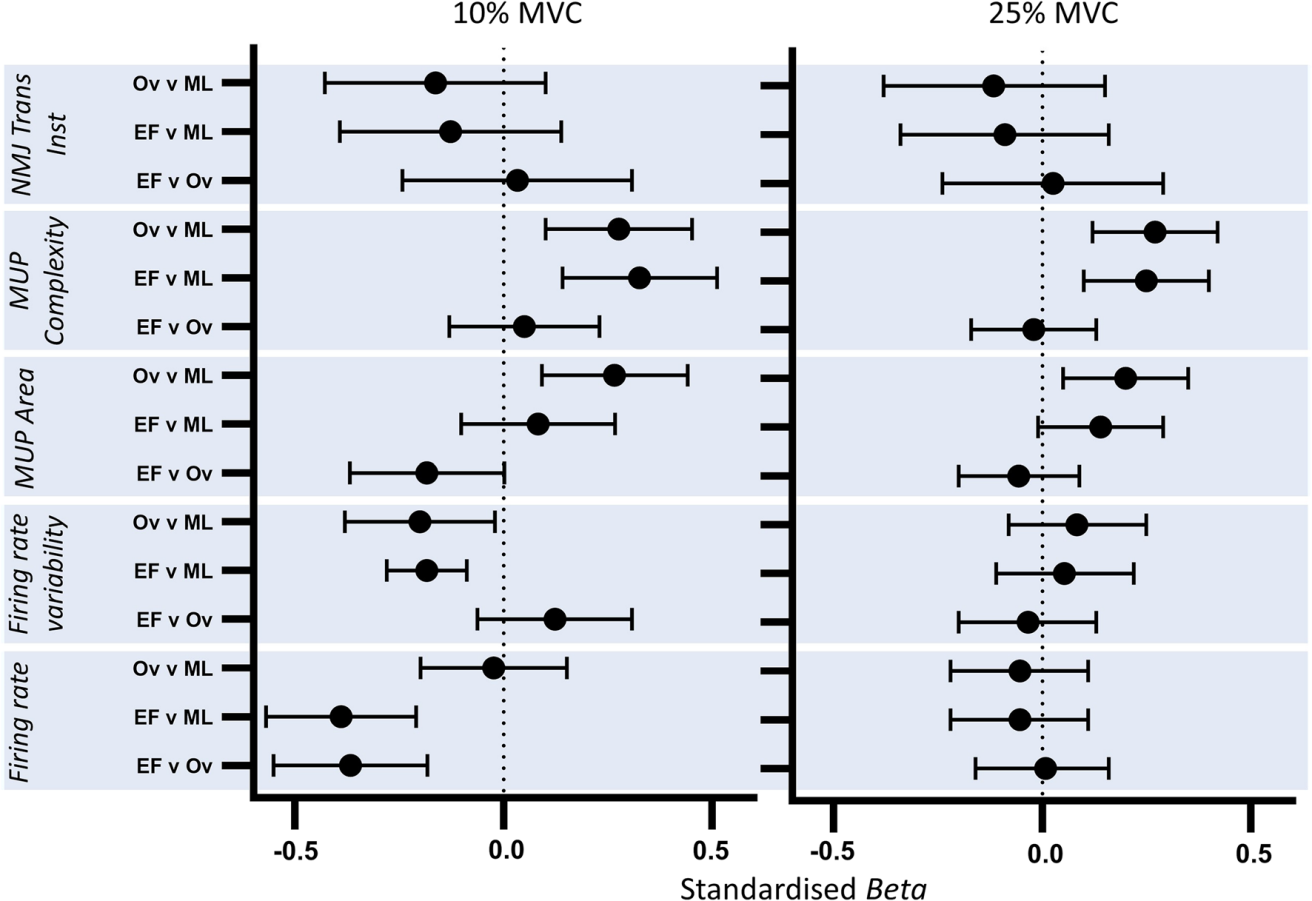
Standardised beta coefficients comparing motor unit parameters recorded during 10% and 25% MVC contractions. Beta value and 95% confidence intervals (CI) represent the standardized model predicted change per unit between menstrual cycle phases. *MUP* Motor unit potential, *NMJ* Neuromuscular junction, *EF* Early follicular, *Ov* Ovulation, *ML* Mid luteal

MUP area measured at 10% MVC did not differ significantly from early follicular to ovulation (*p* = 0.059), or mid luteal (*p* = 0.381), but was larger at mid-luteal compared to ovulation phases (*p* = 0.003). A similar pattern was observed at 25% MVC, with no difference from early follicular to ovulation and mid-luteal (both *p* > 0.06), but a larger MUP area at mid luteal compared to ovulation (*p* = 0.031) (Table 1). MUP complexity, assessed via the number of turns, did not differ from early follicular to ovulation, but was more complex at mid luteal when compared to ovulation (*p* = 0.001) and early follicular (*p* = 0.003) phases. Again, this same pattern was observed at 25% MVC, with no change from early follicular to ovulation (*p* = 0.788), and more complex at mid luteal when compared to ovulation (*p* = 0.001) and early follicular (*p* = 0.001) phases. NMJ transmission instability, measured via NFM jiggle, did not differ at any time point at 10 or 25% MVC (all *p* > 0.3). Individual means and all MUPT data reflecting MUP area, complexity and NMJ transmission instability at 10 and 25% MVC are shown in Fig. 4, with model outputs shown in Table 1. Standardised *β* coefficients of all MUP characteristics recorded at 10% and 25% MVC compared across timepoints are shown in Fig. 5.

**Fig. 6:**
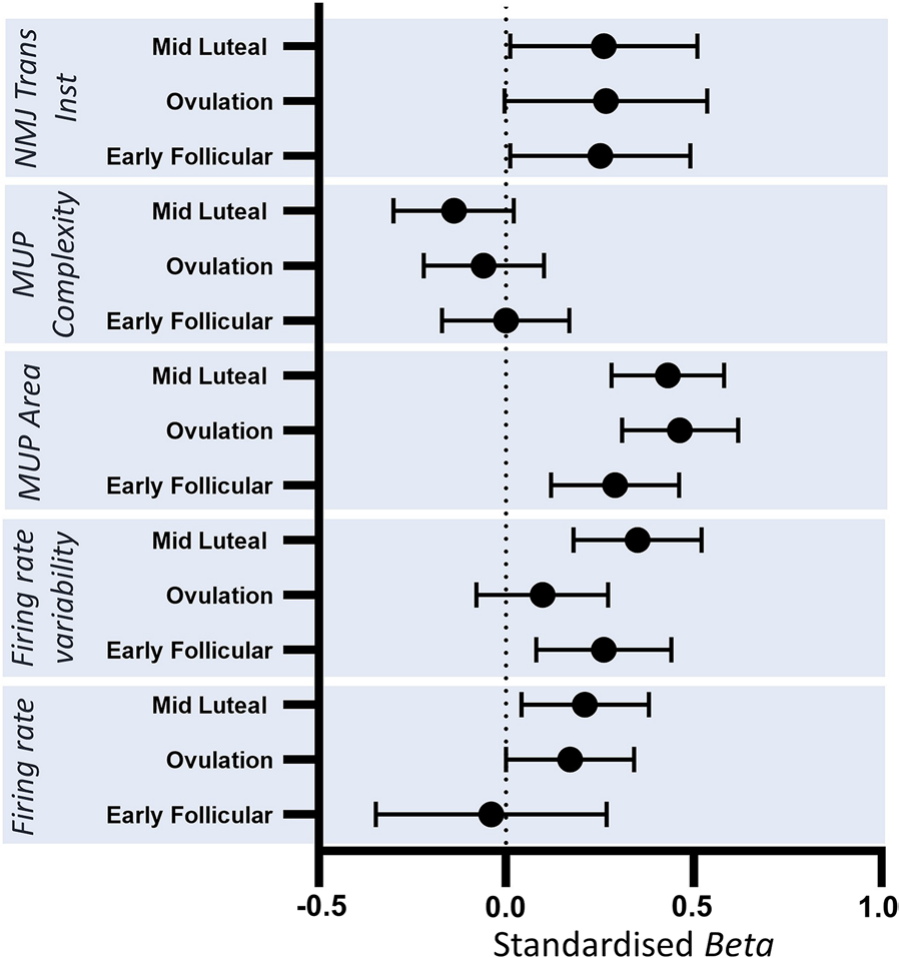
Standardised beta coefficients displaying MUPT parameter differences from 10 to 25% MVC at each timepoint of the cycle. Beta value and 95% confidence intervals (CI) represent the standardized model predicted change per unit between 10 and 25% MVC at each phase of the menstrual cycle. *MUP* Motor unit potential, *NMJ* Neuromuscular junction, *EF* Early follicular, *Ov* Ovulation, *ML* Mid luteal

Recruitment strategies when moving between contraction levels (10 to 25% MVC) at each timepoint in the cycle were also explored. There were no statistically significant differences in MU firing rate at early follicular phase (*p* = 0.179); however firing rate increased from 10 to 25% MVC at ovulation (*p* = 0.047) and mid-luteal phase (*p* = 0.015). There was a significant contraction level x time interaction when comparing the early follicular phase with the ovulation (*p* = 0.09) and the mid luteal phase (*p* = 0.009), indicating a larger contraction level-related increase at these time points (Table 2). Firing rate variability increased in the early follicular (*p* = 0.004) and mid luteal phase (*p* < 0.001), with no change in the ovulation phase (*p* = 0.290) (Table 2). There were no statistically significant contraction level x time interactions in firing rate variability. MUP area increased with increased contraction level at all time points across the cycle (all *p* < 0.002) with no interactions, indicating the degree to which MUP area increased from 10 to 25% MVC did not differ at each timepoint. The number of MUP turns did not differ between 10 and 25% MVC contractions at any time point (all *p* > 0.07). NMJ transmission instability increased with larger contractions at all timepoints (all *p* < 0.049), with no contraction level × time interactions (Table 2). Standardised *β* coefficients of all MUP characteristics displaying relative differences between 10 and 25% MVC at each timepoint are shown in Fig. 6.

**Table 2:** Multilevel model analysis summary comparing motor unit parameters from 10 to 25% MVC, at 3 timepoints of the menstrual cycle

	Beta	95 CI	*p* value
Firing rate (Hz)			
Early follicular	− 0.266	− 0.654 to 0.121	0.179
Ovulation	**0.380**	− **0.010 to 0.770**	**0.047**
Mid luteal	**0.434**	**0.085 to 0.784**	**0.015**
Firing rate variability			
Early follicular	**0.010**	**0.003 to 0.017**	**0.004**
Ovulation	0.004	− 0.003 to 0.010	0.290
Mid luteal	**0.013**	**0.007 to 0.019**	** < 0.001**
MUP area (µV ms)			
Early follicular	**135.21**	**56.47 to 213.95**	**0.001**
Ovulation	**202.84**	**136.48 to 269.21**	** < 0.001**
Mid luteal	**202.65**	**131.92 to 273.38**	** < 0.001**
MUP turns			
Early follicular	− 0.003	− 0.253 to 0.246	0.980
Ovulation	− 0.093	− 0.346 to − 0.716	0.474
Mid luteal	− 0.266	− 0.560 to − 1.769	0.077
NMJ transmission instability (%)			
Early follicular	**2.114**	**0.064 to 4.164**	**0.045**
Ovulation	**2.005**	− **0.087 to 4.096**	**0.049**
Mid luteal	**2.221**	**0.096 to 4.345**	**0.043**

Model outputs show unstandardised beta, 95% confidence interval and associated *p* values

*MVC* Maximum voluntary contraction, *MUP* Motor unit potential, *NMJ* Neuromuscular junction

Bold indicates significantly different

To explore the relationship between each hormone and each mean MUPT parameter across timepoints, regression models with time as a covariate were generated and revealed no significant association between circulating concentrations of estrogen and MUPT parameters measured at 10% or 25% MVC (all *p* > 0.1). There were also no significant associations with progesterone and MUPT parameters measured at 10% and 25% MVC (Table 3).

**Table 3:** Multilevel model analysis summary determining hormone and motor unit parameter associations at 10 and 25% MVC

	10% MVC			25% MVC		
	Beta	95 CI	*p* value	Beta	95 CI	*p* value
Firing rate (Hz)						
Estrogen	0.001	− 0.001 to 0.002	0.363	0.001	− 0.001 to 0.002	0.185
Progesterone	0.027	− 0.164 to 0.218	0.785	− 0.021	− 0.191 to 0.150	0.813
Firing rate variability						
Estrogen	− 0.001	− 0.031 to 0.028	0.923	− 0.011	− 0.035 to 0.012	0.355
Progesterone	− 0.015	− 0.061 to 0.030	0.516	0.035	− 0.002 to 0.072	0.190
MUP area (µV ms)						
Estrogen	− 30.04	− 396.24 to 335.56	0.872	− 130.09	− 649..30 to 387.42	0.625
Progesterone	10.04	− 49.26 to 69.35	0.743	60.14	− 19.66 to 139.95	0.154
MUP turns						
Estrogen	− 0.046	− 0.101 to 0.009	0.545	0.019	− 0.011 to 0.051	0.785
Progesterone	− 0.100	− 0.347 to 0.127	0.373	0.034	− 0.183 to 0.252	0.758
NMJ transmission instability (%)						
Estrogen	0.138	− 0.509 to 0.864	0.718	0.485	− 0.208 to 1.185	0.183
Progesterone	0.430	− 0.706 to 1.567	0.467	0.606	− 0.491 to 1.705	0.291

Model outputs show unstandardised beta, 95% confidence interval and associated *p* values. Time applied as a fixed factor

*MVC* Maximum voluntary contraction, *MUP* Motor unit potential, *NMJ* Neuromuscular junction

## Discussion

This study sought to determine the potential alteration of neuromuscular performance and individual VL MU function across the menstrual cycle in eumenorrheic females and to assess the influence of hormonal fluctuations on these measures. Most notably, low threshold MU firing rate was lower in the latter two phases of the cycle, matched by an increase in MUP size in the final phase which coincided with an increase in circulating progesterone, although statistical outputs show no direct associations between hormone levels and MU parameters. Multiple measures of performance relative to neuromuscular strength and control did not differ at any time point of the cycle, yet the suppression of MU firing rate suggests a neuroinhibitory effect across the latter half of the menstrual cycle preferentially affecting early recruited MUs.

Neuromuscular performance was measured through maximum voluntary contraction, jump power, single leg balance, and force steadiness. Our data did not identify any differences across these measures which aligns with recent reports of very limited performance decrements across the menstrual cycle of eumenorrheic women [46]. Yet it is still quite frequently noted that females report declines in neuromuscular performance through a myriad of symptoms at varying times of the menstrual cycle [56, 57]. Although the functionality data presented herein cannot support this, the range of physiological performance assessments is vast, and decrements may be noted in more prolonged fatiguing assessments [11] than those applied here. Moreover, the large heterogeneity of the menstrual cycle and its effects, reflected in the known inter-individual and intra-individual variations in outcomes of the menstrual cycle (e.g. length, bleeding patterns, symptoms and severity [3, 58, 59]), and a possible disconnect between circulating hormones and their respective functional receptors, may differentially influence outcomes of performance assessments.

MU firing rate at 10% MVC was highest at the early follicular phase (~ 9.77 ± 0.94 Hz) and decreased in ovulation (~ 8.94 ± 0.86 Hz) and mid luteal phases (~ 8.91 ± 1.01 Hz), with no change across the cycle at 25% MVC indicating these effects are contraction level specific. This difference at 10% MVC is comparatively large; it is greater than that we reported in the VL of young males following 15 days of limb immobilisation at normalised and absolute contraction intensities [60] and is larger than that reported following 4-week resistance training in the tibialis anterior [61]. However, in these studies, the neuromodulatory alterations were aligned with changes in force production as a result of the intervention, which was not identified in this female cohort which lacked an intervention. These current findings are also in slight contrast to those of Tenan et al. [10], who reported an increase in firing rate of vastus medialis MUs across five phases of the menstrual cycle quantified via basal body temperature mapping. When comparing the early follicular to the mid-luteal phases, the mean initial firing rate increased from 8.4 to 9.1 Hz in 140 individual MUs recorded from 7 females [10]. Although a different muscle to that assessed here, the VL and Vastus medialis (VM) in young males have similar firing rates [62] and both share common synaptic inputs [63]. It is not entirely clear why the two synergist muscles may be differentially affected by menstrual cycle stage, although some reasoning could be inferred via the number of MUPs within a MUPT used to calculate MU firing rate; all observed MUPs within a MUPT of the sustained plateau of a 12–15 s contraction in the current study, compared to the average of the first 3 MUPs of a ramped contraction [10].

Firing rate is a key determinant of muscle force and typically increases with increased contraction intensity [51, 64]. The difference between 10 and 25% contractions here was more pronounced in the latter phases of the cycle, and although no direct associations were noted between plasma hormone levels and MUP features, these phases were associated with higher progesterone levels. However, these findings also negate the potential net excitatory effects of estrogen [18, 19, 65] and suggest progesterone is the more dominant of the two in this respect. MU firing rate is mediated by ionotropic input from descending pathways, peripheral afferents and spinal interneurons, and via neuromodulatory inputs that can alter the intrinsic excitability of the motoneuron through voltage-dependent ion channels. These latter inputs are known as persistent inward currents (PICs) and act to amplify and prolong synaptic inputs in a nonlinear fashion [66]. Lower threshold MUs are highly sensitive to inhibitory input [67] and it is possible they are more dependent on prolonged PIC activity, which increases with increased contraction level [64], than on descending inputs. As such, they may be more susceptible to the inhibitory effects of progesterone. A variable response of MUs across the entire pool is not uncommon as lower threshold MUs are also more susceptible to FR suppression following disuse [68], and pain induced inhibition [69], although differing to the contraction levels applied here, this likely results from a non-uniform distribution of inhibitory inputs across the MU pool [70]. Firing rate variability partly influences force steadiness [71, 72] and this did not differ across the cycle at either contraction level, aligned with no difference of force steadiness measures.

The area of a MUP typically increases with larger contraction levels as a result of the recruitment of larger MUs [51]. A similar pattern was observed here as increases in MUP area from low to mid-contractions occurred to a similar extent at each phase of the cycle. When comparing across the cycle, MUP area was greatest in the mid luteal phase at both contraction levels. We have previously reported that at a given contraction level, females have smaller MUPs than males but possibly compensate for this with higher comparative firing rates [51], likely mediated by larger PIC estimates [73]. The same may be true here, where MUP area is greater when firing rate is lowest, indicating a minor alteration of recruitment strategy during the mid-luteal phase, compensating for reductions in firing rate at the same relative contraction intensity. However, the difference in MUP area was not observed at the ovulation phase where firing rate was also comparatively lower. We observed a greater number of MUP turns, or MUP complexity in the mid luteal phase where MUP area was also the largest. When the propagation of action potentials along muscle fibres of the same MU are temporally dispersed, the complexity, or number of turns of the recorded MUP, is greater [54]. This can increase acutely in humans following disuse periods of 10 [74] and 15 days [60], occurring in response to pre- and/or post-synaptic mechanisms; a result of temporally dispersed yet consistent transmission at the NMJ of MU fibres, or propagation along MU fibres. Estrogen-induced inhibition of voltage-gated Ca^2+^ channels occurs in smooth muscle [31], and although the current data are far from definitive in this aspect, similar effects at the NMJ could result in increased MUP complexity as transmission at fibres of the same MU becomes more asynchronous. NMJ transmission instability estimated by iEMG and near fibre analysis has consistently shown an increased instability of aged human NMJ transmission [50, 75, 76] and feasibly contributes to age-related decreases in strength. The lack of a difference across the menstrual cycle shown here does not support acute alterations at the NMJ that may be influenced by hormonal fluctuations or other cycle-associated adaptation.

### Strengths and Limitations

To our knowledge, this is the first study to report multiple aspects of neuromuscular and individual MU adaptations of the vastus lateralis muscle across three timepoints of the menstrual cycle with quantification of plasma estrogen and progesterone. The iEMG techniques applied enable identification of adaptations occurring centrally (MU firing rate and firing rate variability) alongside those occurring peripherally at the NMJ and muscle fibre. We applied rigorous methods to accurately quantify the cycle period via self-assessment and hormone quantification and obtained all data within a single cycle to avoid multi-cycle variability. This latter point may be viewed by some as a limitation; all participants completed the three assessments in phase order from the early follicular, to ovulation and finally to the mid-luteal phase which presents the possibility of a learning effect. However, we anticipate this to be minimal given the lack of difference in any performance measure and there were at least seven days between our chosen phases of the cycle, further minimising any possible learning effect.

## Conclusion

Knee extensor muscle force, power, and force steadiness is unaltered across the female menstrual cycle under conditions of fluctuating estrogen and progesterone. There is evidence of adaptation of MU firing rate of low threshold MUs and a probable alteration of recruitment strategy to achieve a given force; however this was not statistically associated with circulating hormone concentrations. NMJ transmission instability was unaltered and highlights minimal effects of the cycle on the peripheral motor system. Although notably interesting from a physiological perspective, these observations had no clear influence on any of the functional assessments of neuromuscular strength and control. Collectively, our human data reveal minimal effects of the menstrual cycle on neuromuscular performance with a probable suppression of firing rate early recruited motor units in the ovulation and mid-luteal phases. These findings offer insight into the functional, or lack thereof, consequences at different stages of the menstrual cycle, where required in research and performance environments, and minimise the perceived limitation of including females in neuromuscular physiology studies.

## Data Availability

The datasets generated and analysed during the current study are available from the corresponding author upon reasonable request.

## Abbreviations

CNS: Central nervous system
COP: Centre of pressure
CoV: Coefficient of variation
DQEMG: Decomposition-based quantitative electromyography
E2: Estradiol
EDTA: Ethylenediamine tetra-acetic acid
EMD: Estimated mean difference
ER: Estrogen receptors
FR: Firing rate
FS: Force steadiness
iEMG: Intramuscular electromyography
ISI: Interspike interval
MU: Motor unit
MUP: Motor unit potential
MUPT: Motor unit potential train
MVC: Maximum voluntary contraction
NFM: Near fibre motor unit potential
NMJ: Neuromuscular junction
ns: Non-significant
PIC: Persistent inward current
SD: Standard deviation
VL: Vastus lateralis
VM: Vastus medialis
